# Experimental and bioinformatics study for production of l-asparaginase from *Bacillus licheniformis*: a promising enzyme for medical application

**DOI:** 10.1186/s13568-019-0751-3

**Published:** 2019-03-21

**Authors:** Nada A. Abdelrazek, Walid F. Elkhatib, Marwa M. Raafat, Mohammad M. Aboulwafa

**Affiliations:** 1 Department of Microbiology and Immunology, Faculty of Pharmaceutical Sciences and Pharmaceutical Industries, Future University, Cairo, Egypt; 2 Department of Microbiology and Immunology, Faculty of Pharmacy, Ain Shams University, African Union Organization St. Abbassia, Cairo, 11566 Egypt; 3 Department of Microbiology and Immunology, School of Pharmacy & Pharmaceutical Industries, Badr University in Cairo (BUC), Entertainment Area, Badr, Cairo Egypt

**Keywords:** l-Asparaginase, *Bacillus licheniformis*, Response surface methodology, Optimization, Characterization, Bioinformatics

## Abstract

A *Bacillus licheniformis* isolate with high l-asparaginase productivity was recovered upon screening two hundred soil samples. This isolate produces the two types of bacterial l-asparaginases, the intracellular type I and the extracellular type II. The catalytic activity of type II enzyme was much higher than that of type I and reached about 5.5 IU/ml/h. Bioinformatics analysis revealed that l-asparaginases of *Bacillus licheniformis* is clustered with those of *Bacillus subtilis*, *Bacillus haloterans*, *Bacillus mojavensis* and *Bacillus tequilensis* while it exhibits distant relatedness to l-asparaginases of other *Bacillus subtilis* species as well as to those of *Bacillus amyloliquefaciens* and *Bacillus velezensis* species. Upon comparison of *Bacillus licheniformis* l-asparaginase to those of the two FDA approved l-asparaginases of *E. coli* (marketed as Elspar) and *Erwinia chrysanthemi* (marketed as Erwinaze), it observed in a cluster distinct from- and with validly predicted antigenic regions number comparable to those of the two mentioned reference strains. It exhibited maximum activity at 40 °C, pH 8.6, 40 mM asparagine, 10 mM zinc sulphate and could withstand 500 mM NaCl and retain 70% of its activity at 70 °C for 30 min exposure time. Isolate enzyme productivity was improved by gamma irradiation and optimized by RSM experimental design (Box–Behnken central composite design). The optimum conditions for maximum l-asparaginase production by the improved mutant were 39.57 °C, 7.39 pH, 20.74 h, 196.40 rpm, 0.5% glucose, 0.1% ammonium chloride, and 10 mM magnesium sulphate. Taken together, *Bacillus licheniformis* l-asparaginase can be considered as a promising candidate for clinical application as antileukemic agent.

**Electronic supplementary material:**

The online version of this article (10.1186/s13568-019-0751-3) contains supplementary material, which is available to authorized users.

## Introduction

Enzymes play an important role in metabolic and biochemical reactions and microorganisms are the primary source (Nigam 2013), as they can be cultured in large quantities in short span of time (Anbu et al. 2013; Gopinath et al. 2013). l-Asparaginase is a therapeutic enzyme which has proved to be promising for the treatment of acute lymphocytic leukemia (Sinha et al. 2013). Unlike normal cells, malignant cells can only slowly synthesize l-asparagine, due to their deficiency in l-asparagine synthetase. Thus depletion of the circulating pools of l-asparagine by l-asparaginase leads to the destruction of the tumor cells, since they are unable to complete protein synthesis by inhibition of RNA and DNA synthesis with subsequent blastic cell apoptosis (Bansal et al. 2012). l-Asparaginase has been introduced into the pretreatment of potato slices and bread dough before frying or baking to prevent acrylamide formation (carcinogenic toxicant) (Krishnapura et al. 2016). Also, this enzyme acts as a biosensor to detect the amount of asparagine in leukemia and food industry (Batool et al. 2016). The current study used bioinformatics and experimental approaches for production and characterization of l-asparaginase from the recovered soil isolate, *Bacillus licheniformis*. The study gives evidence for the introduction of *Bacillus licheniformis* l-asparaginase as a potentially comparable and additional source to those of the two FDA approved ones from *E. coli* (marketed under the brand name Elspar) and *Erwinia chrysanthemi* (marketed under the brand name Erwinaze) to be used as antileukemic agent.

## Materials and methods

### Chemicals

All chemicals were supplied, unless otherwise indicated, by El-Nasr chemicals ADWIC (Cairo, Egypt). l-Asparagine monohydrate was product of AppliChem GmbH (Darmstadt, Germany).

### Bacterial strain and maintenance

*Bacillus licheniformis* isolate was obtained from screening of 722 soil isolates for l-asparaginase production.

### Isolation and qualitative detection of l-asparaginase production by recovered soil bacteria

This was principally carried out according to Izadpanah et al. (2014). This method depends on the appearance of pink zone around l-asparaginase producing colonies on modified M9 agar medium containing 1% w/v asparagine and phenol red as an indicator.

### Inoculum preparation and l-asparaginase production

The inoculum was prepared by inoculating 20 ml modified M9 broth contained in 250 ml Erlenmeyer flask with single isolated colony. The flask was incubated at 37 °C and 180 rpm for 24 h. The broth culture obtained was diluted by fresh M9 broth medium to an O.D. = 1.0 at 600 nm to be used as an inoculum. The enzyme production was carried out in 250 ml Erlenmeyer flasks, the flasks were inoculated with 2% v/v from the cell suspension (Mahajan et al. 2012) and incubated at 37 °C and 180 rpm for 24 h. An aliquot (2 ml) of the broth culture obtained was centrifuged at 4 °C and 5000 rpm for 20 min using cooling centrifuge (Jain et al. 2012). The produced supernatant was termed crude enzyme preparation and used for quantitative assay of extracellular l-asparaginase while the produced pellets were lysed and tested for any intracellular enzyme activity. For the preparation of crude cell lysate, the cell pellets were washed twice with 50 mM Tris–HCl (pH 7.5) and suspended in 30 ml lysis buffer (Straight et al. 2007). Cells were then disrupted by sonication using sonication probe under cooling condition at 4 °C. Cellular debris and unbroken cells were removed by centrifugation at 15,000 rpm and 4 °C for 15 min (Sakr et al. 2014) and the supernatant was collected for determination of intracellular enzyme activity.

### Quantitative assay of l-asparaginase

l-Asparaginase activity was measured by the method described by of Mashburn and Wriston (1963). The assay depends on hydrolysis of l-asparagine by the enzyme preparation to release ammonia. One unit of l-asparaginase activity is defined as the amount of enzyme required for the release of one micromole of ammonia per hour at 37 °C and pH 8.6 (Mahajan et al. 2014).

### Identification of soil isolate with highest l-asparaginase productivity

The isolate of the highest l-asparaginase productivity was identified by microscopical examination (Gram stain), biochemical reactions (using Biolog^®^ system) and confirmed by the 16S rRNA gene sequencing.

### Bioinformatics analysis

The degrees of relatedness of *Bacillus licheniformis* l-asparaginase to other microbial l-asparaginases (the amino acid sequence of the enzyme of that organism was used as a probe to retrieve NCBI database similar sequences in BLAST) and to the two FDA approved l-asparaginases of *E. coli* (marketed under the brand name Elspar) and *Erwinia chrysanthemi* (marketed under the brand name Erwinaze) were inferred by the Maximum Likelihood method based on the JTT matrix-based model (Jones et al. 1992) using their amino acid sequences. The phylogenetic tree was drawn to scale, with branch lengths measured in the number of substitutions per site. Evolutionary analyses were conducted in MEGA X (Kumar et al. 2018). The antigenic sites in *Bacillus licheniformis* l-asparaginase as compared to those in the two FDA approved l-asparaginases of *E. coli* and *Erwinia chrysanthemi* were predicted using the method of Kolaskar and Tongaonkar (1990) (EMBOSS: antigenic—Bioinformatics web site http://www.bioinformatics.nl/cgi-bin/emboss/antigenic).

### l-Asparaginase characterization

The crude preparation of l-asparaginase (supernatant of growth culture) of the selected isolate was evaluated for different characteristics of industrial importance which included thermal stability, activity at different temperatures, pH values, salinities, substrate concentrations, and metal ions.

### Improvement of l-asparaginase production of the selected isolate by mutation with gamma irradiation

Five ml aliquot of a prepared spore suspension (Seale et al. 2008), contained in 10 ml sterile screw capped glass tubes, was exposed to different doses of gamma rays 0.1, 0.5, 1, 3, and 5 KGy (Diep et al. 2017). After irradiation, a number of recovered colonies were selected randomly to be qualitatively and quantitatively assessed for l-asparaginase production in comparison to the parent wild strain. The mutant with the highest l-asparaginase productivity was selected for completing the present study.

### Effect of different environmental and physiological factors influencing l-asparaginase production by the selected mutant

Different environmental factors including incubation temperature, initial pH, incubation time, agitation rate as well as various media components were evaluated for their effects on l-asparaginase production. In all cases, at the end of the incubation period l-asparaginase activity was quantitatively determined as described before except that in case of studying the effect of incubation time where samples were removed at different time intervals for l-asparaginase activity measurements.

### Optimization of l-asparaginase production using response surface methodology (RSM) experimental design

From preliminary conducted studies, four process parameters [incubation temperature coded (A), pH values coded (B), incubation time coded (C) and agitation rate coded (D)] were optimized by RSM experimental design (Box–Behnken central composite design). Each parameter was examined at 3 levels that correspond to the 3 highest l-asparaginase productivity obtained. The mean level of each parameter was coded (0) and it represents the average of the 2 levels that showed the highest and the lowest l-asparaginase production. The maximum level coded (+ 1) and the lower level coded (− 1). The range of studied variables is shown in Additional file 1: Table S1. Design-Expert 7 (Stat-Ease Inc., Minneapolis, MN, USA) was used for experimental design as well as graphical analyses of the data and regressions. A number of 27 experiments were obtained. The codes and values of the three levels for the studied variables (n = 4) are shown in Additional file 1: Table S2. These experiments were principally carried out as mentioned before, except that the environmental conditions (incubation temperature, initial pH, incubation time, agitation) were set at the values listed in Additional file 1: Table S2. The results obtained from the 27 experiments were analyzed by the used software to determine the response surface contour plots, the regression equation and the test variables optimum levels.

### Effect of different media components

The effect of different carbon sources (glucose, sucrose, fructose, lactose, maltose, glycerol, starch and arabinose), nitrogen sources (ammonium chloride, potassium nitrate, ammonium nitrate, yeast extract, peptone, urea, tryptone) and metal ions (copper sulphate, calcium chloride dihydrate, magnesium sulphate heptahydrate, cobalt chloride, manganese sulphate zinc sulphate heptahydrate) were evaluated for their effects on l-asparaginase production by the test mutant. Each of the carbon source, the nitrogen source and the metal ion source that showed maximum l-asparaginase productivity was re-tested at different concentrations.

### Statistical and graphical analyses

All experiments were carried out in triplicates and the mean as well as standard deviation were calculated. The data were statistically analyzed using one way ANOVA followed by Dunnett’s Multiple Comparison Test. All tests were performed using Graph Pad Prism Version 5.0 (GraphPad Software, La Jolla, CA, USA).

## Results

### Recovery and identification of a promising l-asparaginase producing isolate

A promising l-asparaginase-producing bacterial isolate was selected using an extensive screening program on 722 recovered bacterial soil isolates. This isolate was identified using the methods listed in materials and methods as *Bacillus licheniformis* and its 16S rRNA gene sequence was deposited in GenBank database under the accession number MG665995, the strain also deposited in Egypt Microbiological Culture collection (EMCC) with number EMCC 2290. The extracellular l-asparaginase productivity of this test isolate exceeded the intracellular one by at least threefolds (data not shown). Accordingly, l-asparaginase characterization and production optimization were based on the extracellular enzyme productivity.

### Bioinformatics analysis

**Fig. 1:**
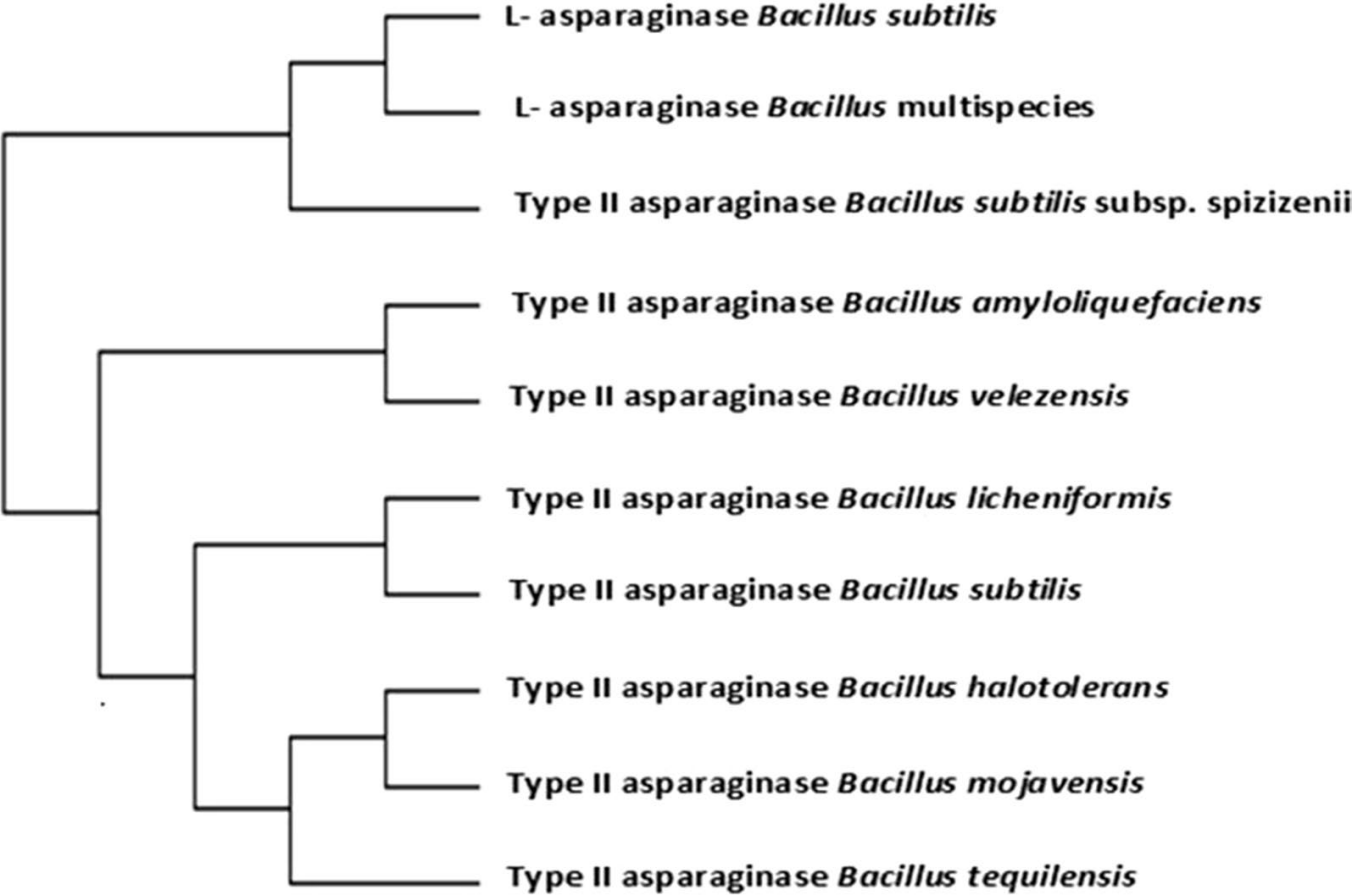
Molecular phylogenetic analysis by maximum likelihood method of *Bacillus licheniformis* l-asparaginase (accession WP_075750324) as compared to those retrieved by blasting the query sequence against amino acid sequences deposited in NCBI databases. l-asparaginases presented in the tree are non-redundant ones with amino acids sequence similarities not less than 74%

**Fig. 2:**
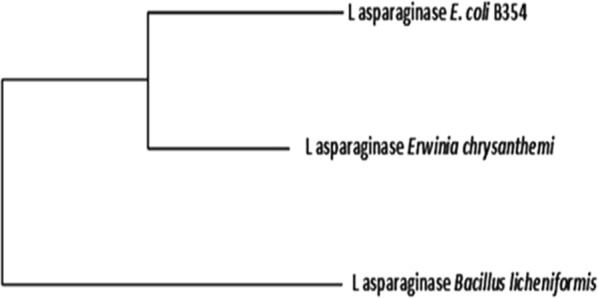
Molecular phylogenetic analysis by maximum likelihood method of *Bacillus licheniformis* l-asparaginase (accession WP_075750324) when blasted against amino acid sequences of the FDA approved l-asparaginases of *E. coli* and *Erwinia chrysanthemi*

The molecular phylogenetic tree of l-asparaginases with amino acids sequence similarities not less than 74% to the target query l-asparaginase of *Bacillus licheniformis* is shown in Fig. 1 while that of the target query l-asparaginase and the two FDA approved l-asparaginases of *E. coli* (marketed under the brand name Elspar) and *Erwinia chrysanthemi* (marketed under the brand name Erwinaze) is shown in Fig. 2. The corresponding pairwise distances among l-asparaginases for bacterial species presented in Fig. 1 is shown in Table (S3) while those for l-asparaginases of bacterial species presented in Fig. 2 is illustrated in Additional file 1: Table S4. Potentially antigenic regions of a l-asparaginase sequence of *Bacillus licheniformis* were predicted and compared to those determined for *E. coli* and *Erwinia chrysanthemi* using the prediction program EMBOSS antigenic explorer^®^ as mentioned in Materials and Methods. The results presented in Table 1 reveal 18, 16 and 17 antigenic regions, their positions and sequences for l-asparaginases of *E. coli*, *Erwinia chrysanthemi* and *Bacillus licheniformis*, respectively.

**Table 1:** Antigenic regions, their positions, and sequences of *E. coli, Erwinia chrysanthemi*, and *Bacillus licheniformis* l-asparaginases

*E. coli* l-asparaginase (348 aa) Accession # D6JF83^a^				*Erwinia chrysanthemi* l-asparaginase (348 aa) Accession # P06608^b^				*Bacillus licheniformis* l-asparaginase (322 aa) Accession # P30363^c^			
Antigenic region	Start	End	Sequence	Anti-genic region	Start	End	Sequence	Anti-genic region	Start	End	Sequence
1	323	333	KARVLLQLALT	1	6	20	KSLFVLVLFFVFTS	1	195	216	KRDVHQLQRPLPAVDIVKCYLD
2	224	243	PFDVSKLNELPKVGIVYNYA	2	121	142	ESAYFLHLTVKSDKPVVFVAAM	2	304	314	KLAVLLASYKE
3	245	257	ASDLPAKALVDG	3	88	101	GDVVLKLSQRVL	3	256	265	NQGVYIVITT
4	118	135	AYFLDLTVKCDKPVVMVG	4	290	297	KGVVVIRS	4	128	140	NIRHAVYTACSPD
5	6	16	KTALAALVMGF	5	156	165	LLEAVRVAGD	5	184	193	NDKVYVYQKP
6	165	174	NRGVLVVMND	6	311	333	LPGLVSDSLNPAHARILLMLALT	6	144	152	AGTVVVFNE
7	49	70	AGKVGVENLVNAVPQLKDIANV	7	22	34	ADKLPNIVILATG	7	35	58	AEMCSLPEDVQIDVYPAFQLPSMH
8	151	159	LYNAVVTAA	8	172	179	GVMVVLND	8	106	114	ERPVVVTGS
9	286	298	GTAVVRSSRVPTG	9	107	114	VDGVVITH	9	157	168	ARYVKKVHASNL
10	72	79	EQVVNIG	10	251	270	PEYLYDAAIQHGVKGIVYAG	10	231	237	EGIVLEG
11	190	204	VATFKSVNYGPLGYI	11	226	248	TRSVFDVRGLTSLPKVDILYGYQ	11	170	181	GFDVFGFGYLGI
12	259	265	DGIVSAG	12	205	214	YLGVIIGNRI	12	95	102	TAYFLDLT
13	88	94	WLTLAKK	13	52	73	AGALGVDTLINAVPEVKKLANV	13	60	68	TFEHLLELK
14	18	32	GAALALPNITILAG	14	337	345	DPKVIQEYF	14	81	87	DGAVVTH
15	272	282	YKTVFDTLATA	15	274	281	GSVSVRGI	15	4	9	KVALIT
16	311	317	YGFVASG	16	303	309	GIVPPDE	16	278	294	YAGSSYDLAKKGVILGK
17	339	345	QQIQQIF					17	24	29	LAAGAI
18	104	109	GFVITH								

^a^*E. coli* FDA approved l-asparaginase (marketed under the brand name Elspar)

^b^*Erwinia chrysanthemi* (*Dickeya chrysanthemi* or *Pectobacterium chrysanthemi*) l-asparaginase (marketed under the brand name Erwinaze)

^c^*Bacillus licheniformis* l-asparaginase

### l-Asparaginase characterization of the test isolate

**Fig. 3:**
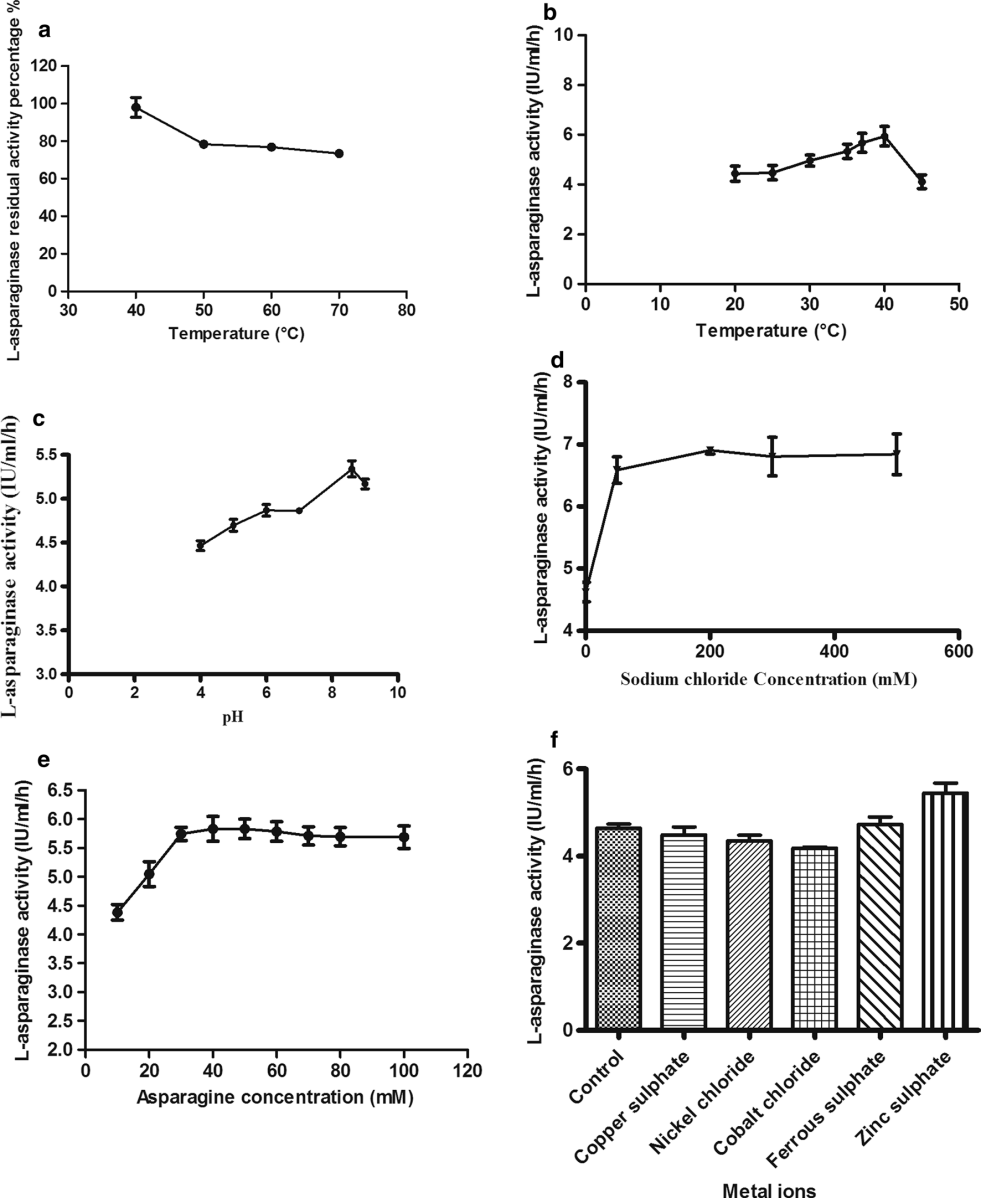
Thermal stability (**a**) and catalytic activities at different temperatures (**b**), pH values (**c**), salinities (**d**), substrate concentrations (**e**) and metal ions (**f**) of l-asparaginase produced by *Bacillus licheniformis*

The results (Fig. 3) revealed that l-asparaginase activity was not dramatically affected when exposed to temperature up to 50 °C for 30 min, and the maximal activity was observed at 40 °C, pH 8.6 and 40 mM asparagine concentration. The enzyme activity increased by incorporation of sodium chloride up to 100 mM and the enzyme could efficiently retain its activity at high salinity up to 500 mM sodium chloride. From the tested metal salts (copper sulphate, nickel chloride, cobalt chloride, ferrous sulphate and zinc sulphate), zinc sulphate was the only metal salt that showed significant (*p *<* 0.05*) increase in enzyme activity. Other tested metal salts showed no significant effect as compared to the control.

### Strain improvement

Gamma irradiation was utilized to improve l-asparaginase production by the test isolate. The results revealed that exposure to 5 KGy gamma radiations enabled the selection of a mutant with higher l-asparaginase productivity compared to the lower tested doses of gamma radiations (data not shown). The enzyme productivity of the selected mutant was 1.4 fold higher than that of the parent strain.

### Model-based optimization of l-asparaginase production by *Bacillus licheniformis* mutant

#### Effect of environmental conditions and RSM experimental design

**Fig. 4:**
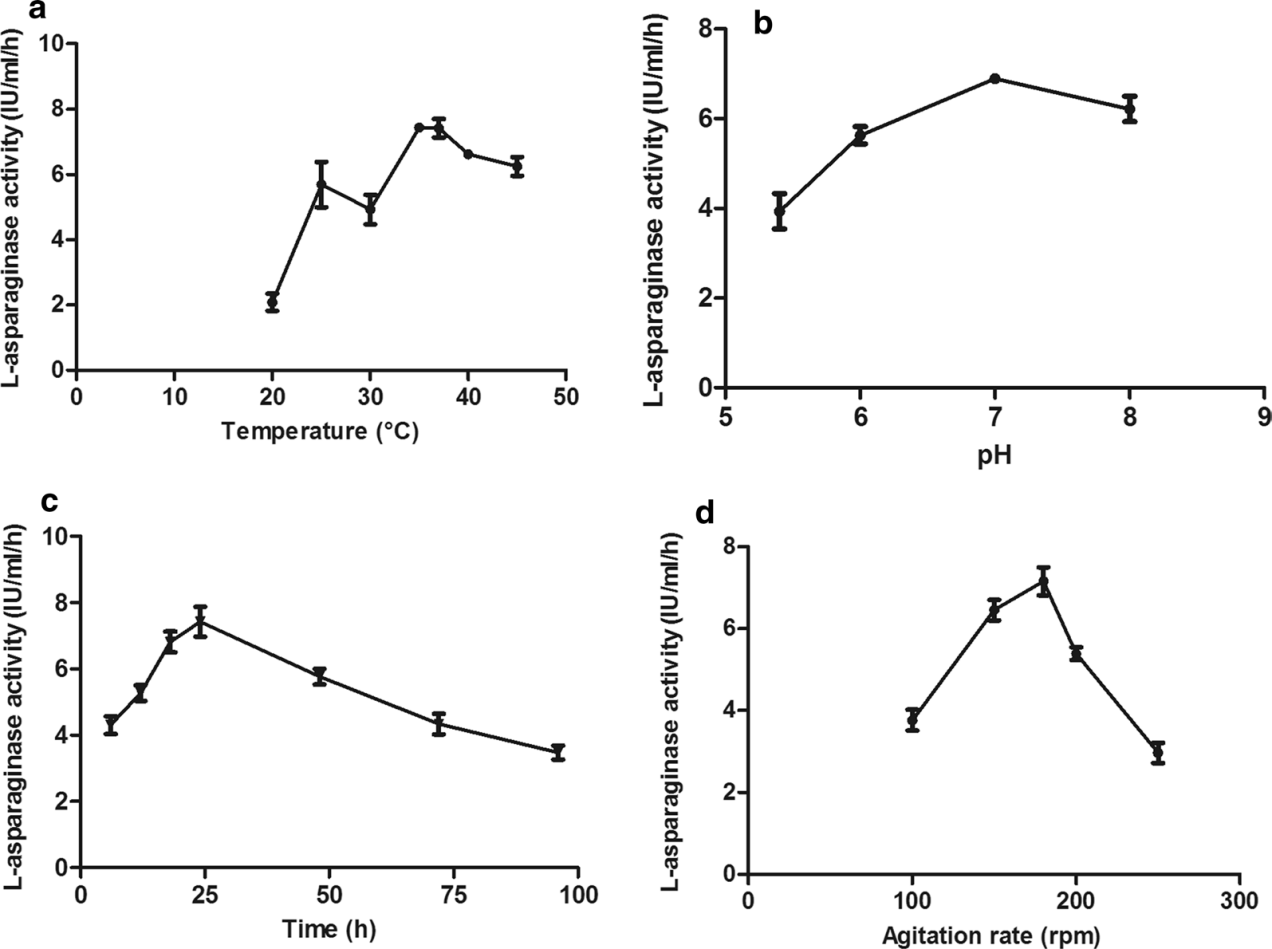
Effect of incubation temperature (**a**), initial pH (**b**), incubation time (**c**), and agitation rate (**d**) on l-asparaginase production by *Bacillus licheniformis* mutant. The enzyme productivity was expressed in terms of catalytic activity

Regarding the effect of incubation temperature, it was found that low level of l-asparaginase production by *Bacillus licheniformis* mutant occurs at 20 °C, reached its maximum level at 37 °C and slightly decreased thereafter up to 50 °C. Concerning the effect of initial pH, there was a considerable l-asparaginase production at all the tested pH values with a maximum productivity achieved at pH 7. Regarding the effect of incubation time, the results revealed that the maximum l-asparaginase production by the test mutant is attained at 24 h followed by gradual decrease in the production. In case of agitation rate, the maximum l-asparaginase productivity was obtained at 180 rpm and lower productivities at agitation rates around this value were noted (Fig. 4). RSM experimental design was applied on pretested environmental parameters (four variables) which included incubation temperature, initial pH, incubation time and agitation rate. The results of the response surface model including observed, predicted, residual values are given in Table 2. According to the mathematical model regression equation, the predicted values were calculated as follows:

$$ \begin{aligned} {\text{Sqrt }}\left( \textsc{l}{\text{-asparaginase activity}} \right) \, & = \, + 2. 6 8 + \, 0.0 1 4*{\text{A }} + \, 0.0 5 6*{\text{B }} - \, 0. 1 4*{\text{C }} + \, 0.0 2 4*{\text{D}}  \\ \, & -{ 2}. 1 2 7 {\text{E}} - 00 4*{\text{A}}*{\text{B }} + \, 0.0 1 3*{\text{A}}*{\text{C }} + \, 0.0 3 3*{\text{A}}*{\text{D }} \\ & - \, 0.0 1 3*{\text{B}}*{\text{C }} + \, 0.0 1 7*{\text{B}}*{\text{D }} + \, 0.0 1 7*{\text{C}}*{\text{D }} \\ & {-}{ 2}. 4 10{\text{E}} - 00 4*{\text{A}}^{ 2} - \, 0. 100*{\text{B}}^{ 2} - \, 0.0 1 7*{\text{C}}^{ 2} - \, 0.0 1 8*{\text{D}}^{ 2} \\ \end{aligned} $$

where l-asparaginase activity was expressed in square root values, and A, B, C and D represent incubation temperature, initial pH value, incubation time and agitation rate, respectively.

**Table 2:** Observed, predicted, and residual values for process parameters optimization of l-asparaginase productivity by *Bacillus licheniformis* mutant using Box–Behnken central composite design

Experiment	Temperature (°C)	pH	Time (h)	Agitation (rpm)	Square root of l-asparaginase activity (IU/ml/h)		Residual value
					Observed value	Predicted value	
1	35	6	33	175	2.52	2.51	0.01
2	40	6	33	175	2.49	2.54	− 0.05
3	35	8	33	175	2.67	2.63	0.04
4	40	8	33	175	2.64	2.65	− 0.01
5	37.5	7	18	150	2.76	2.78	− 0.02
6	37.5	7	48	150	2.45	2.47	− 0.02
7	37.5	7	18	200	2.81	2.79	0.02
8	37.5	7	48	200	2.56	2.55	0.01
9	35	7	33	150	2.66	2.66	0
10	40	7	33	150	2.65	2.62	0.03
11	35	7	33	200	2.61	2.64	− 0.03
12	40	7	33	200	2.74	2.74	0
13	37.5	6	18	175	2.65	2.62	0.03
14	37.5	8	18	175	2.79	2.77	0.02
15	37.5	6	48	175	2.37	2.39	− 0.02
16	37.5	8	48	175	2.46	2.47	− 0.01
17	35	7	18	175	2.76	2.8	− 0.04
18	40	7	18	175	2.8	2.8	0
19	35	7	48	175	2.52	2.5	0.02
20	40	7	48	175	2.6	2.56	0.04
21	37.5	6	33	150	2.53	2.5	0.03
22	37.5	8	33	150	2.57	2.58	− 0.01
23	37.5	6	33	200	2.54	2.52	0.02
24	37.5	8	33	200	2.65	2.66	− 0.01
25*	37.5	7	33	175	2.67	2.68	− 0.01
26*	37.5	7	33	175	2.69	2.68	0.01
27*	37.5	7	33	175	2.68	2.68	0

* The 3 replicates at the center point of the tested values (pH, temperature, time, agitation)

Additional file 1: Table S5 shows ANOVA of the obtained quadratic model. Model F-value of 19.59 implies that the model is significant. For the obtained F-value, a p-value less than 0.0001 means that there is only 0.01% chance that this large model F-value could occur due to noise. The regression coefficient of the model (R^2^) was evaluated to test the fit of the model. The R^2^ was calculated to be 0.9581, referring that the model could explain 95.81% of the variability. Only 4.19% of the total variation is not explained by the model. The “Predicted R-Squared” of 0.7600 is in reasonable agreement (a difference not exceeding the recommended value of 0.3 with the “Adjusted R-Squared” of 0.9092 (Frost 2013).

The adequate precision measures the signal (response) to noise ratio. It is desirable that the ratio to be greater than 4. A ratio 15.913 indicates an adequate signal to noise ratio. Therefore this model can be used to navigate the design space. The “lack of fit” used to compare the residual error to the “pure error” from replicated design points. The “lack of fit F-value” of 19.2 implies that the lack of fit is not significant relative to the pure error. Non-significant lack of fit indicates the model fits. As the magnitude of F-value became larger and the magnitude of *p* value smaller, the corresponding coefficient is more significant (Adinarayana and Ellaiah 2002). Table 3 lists the process parameters that proved to be significant for l-asparaginase productivity.

**Table 3:** Process parameters having significant effects on l-asparaginase productivity by *Bacillus licheniformis* shown in descending order of significance

Order	Process parameter factor
1	Incubation time
2	Square term of initial pH
3	Initial pH
4	Agitation rate

#### Graphical representation of dual interactions of process parameters

**Fig. 5:**
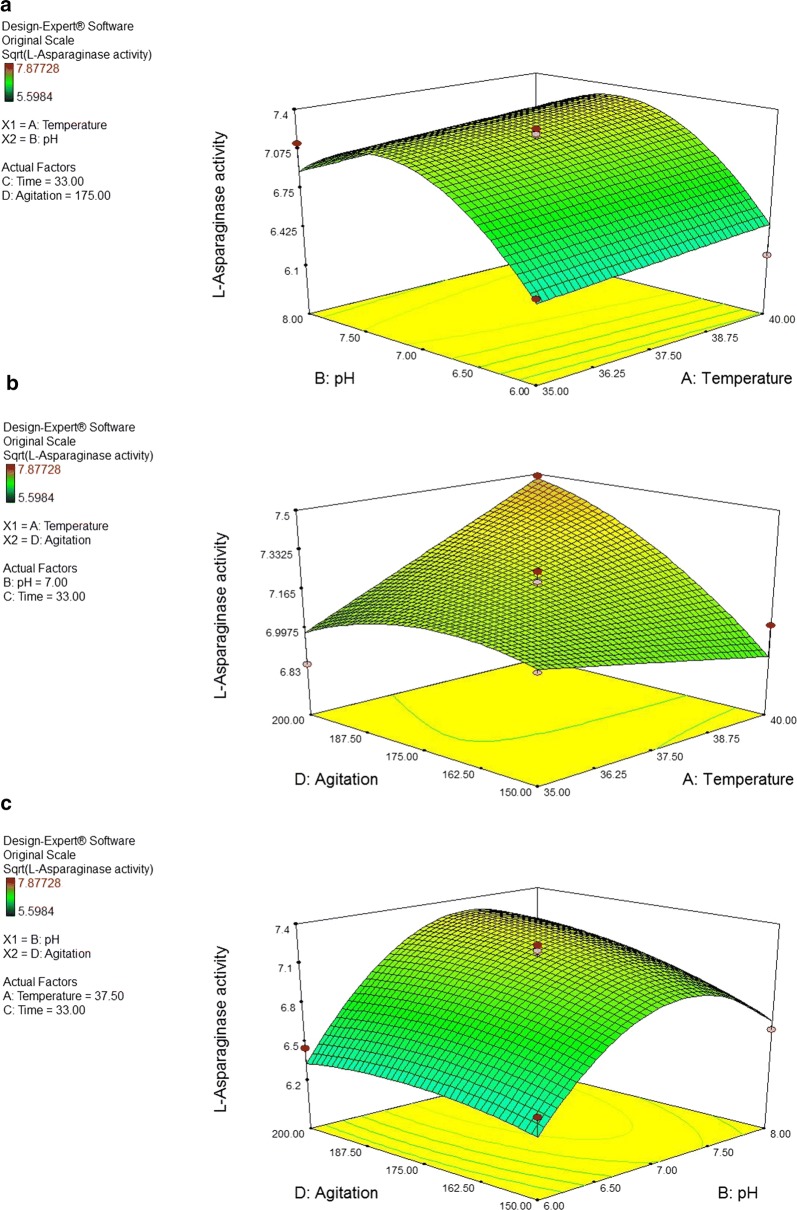
Response surface plots for the optimization of process parameters showing the effect of **a** the interaction between temperature (°C) and pH; **b** the interaction between temperature (°C) and agitation (rpm); **c** the interaction between pH and agitation (rpm) on l-asparaginase production by *Bacillus licheniformis* mutant strain. The enzyme productivity was expressed in terms of catalytic activity

**Fig. 6:**
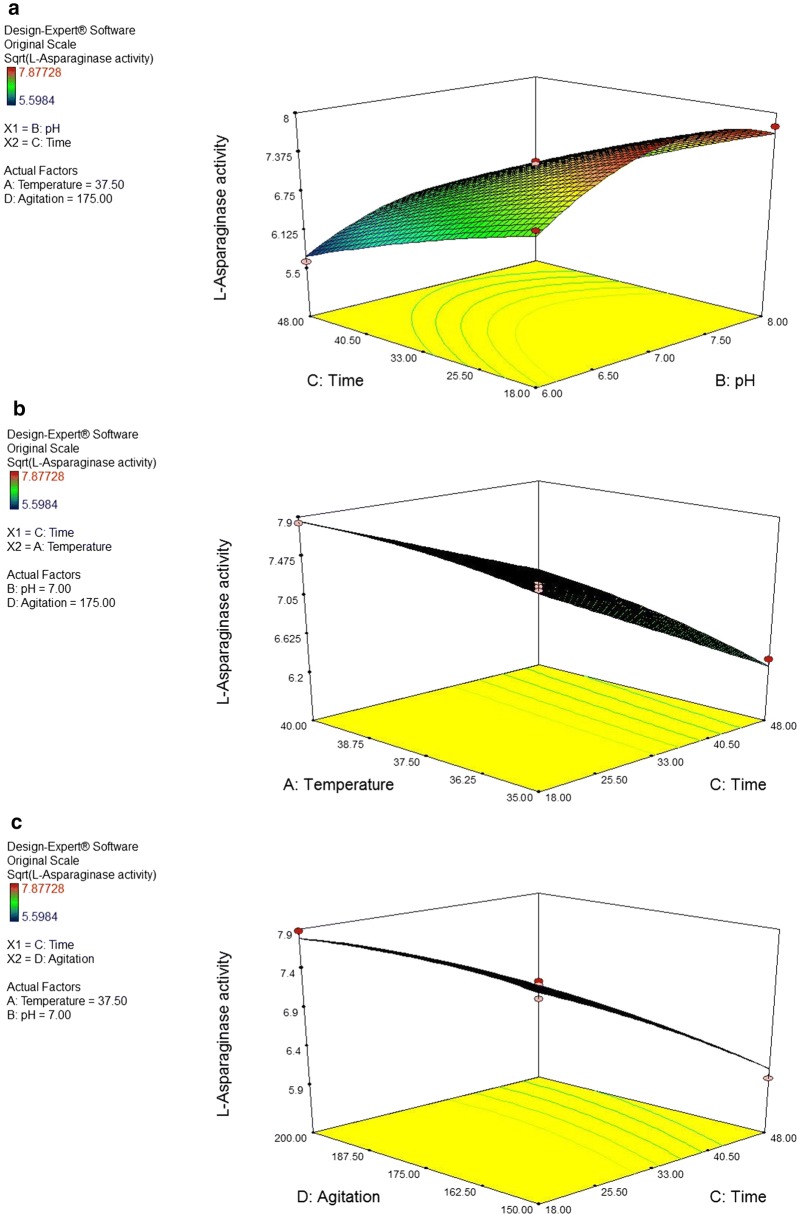
Response surface plots for the optimization of process parameters showing the effect of **a** the interaction between pH and time (h); **b** the interaction between time (h) and temperature (°C); and **c** the interaction between time (h) and agitation (rpm) on l-asparaginase production by *Bacillus licheniformis* mutant strain. The enzyme productivity was expressed in terms of catalytic activity

The 3D response surface and 2D contour plots (Figs. 5, 6) are the graphical representations of the regression equation. Response surface plots [RSPs] (a) and (b) show the effect of temperature and its interactions with pH and agitation, respectively. The interactions reveal the predicted maximum l-asparaginase productivity of 7.30761 IU/ml/h at pH 7.19 and temperature 39.99 °C and 7.48121 IU/ml/h at the same temperature (39.99 °C) 200 rpm. On the other hand, RSPs (c) and (d) demonstrate the effect of pH and its interactions with agitation and incubation time, respectively. The interactions show the predicted maximum l-asparaginase productivity of 7.3004 IU/ml/h at pH 7.35 and agitation 199.95 rpm and 7.90794 IU/ml/h at pH 7.46 and 18.01 h. RSPs (e) and (f) represent the effect of incubation time and its interactions with temperature and agitation rate, respectively. Their interactions also predict maximum l-asparaginase productivity of 7.85147 IU/ml/h at temperature 40 °C and time 18 h and of 7.8464 IU/ml/h at the same incubation time (18 h) and 184.44 rpm.

The main objective of applying response surface methodology is to specify the optimum value of each variable to maximize the studied response. According to the applied model, the predicted maximum value of l-asparaginase productivity of *Bacillus licheniformis* is 7.9518 IU/ml/h and it can be obtained at temperature of 39.5 °C, pH of 7.4, incubation time of 21 h, and agitation rate of 196 rpm.

#### Effect of media components

**Fig. 7:**
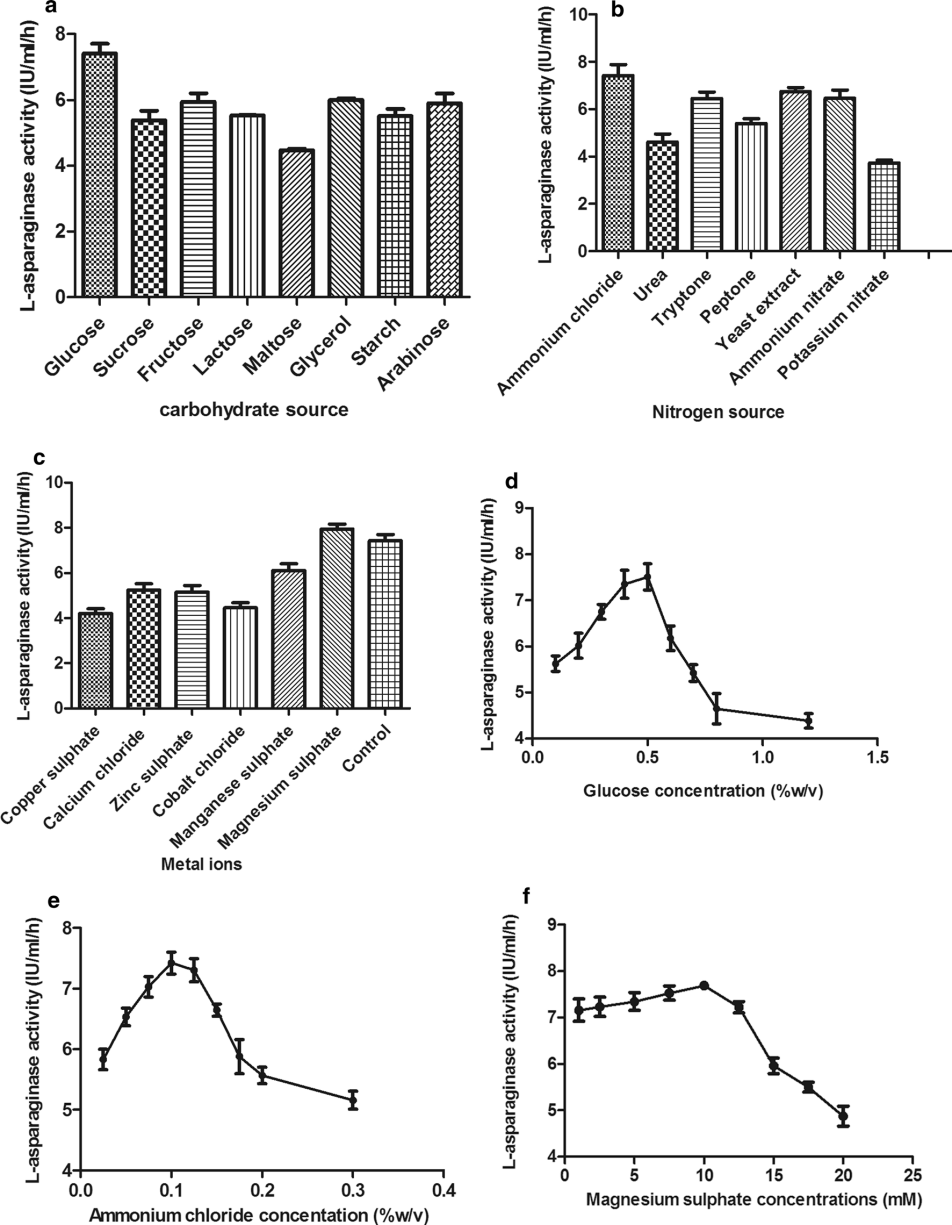
Effect of different carbon sources (**a**), nitrogen sources (**b**), metal ions (**c**) and different concentrations of glucose (**d**), ammonium chloride (**e**) and magnesium sulphate (**f**) on l-asparaginase production by *Bacillus licheniformis* mutant strain. The enzyme productivity was expressed in terms of catalytic activity

By studying the effect of different carbon sources (glucose, sucrose, fructose, lactose, maltose, glycerol, starch and arabinose), glucose proved to be the best carbon source for l-asparaginase production. Regarding the nitrogen sources, ammonium chloride showed the highest enzyme production. Furthermore, incorporation of different metal ions into the culture medium revealed that the highest enzyme productivity occurred with magnesium sulphate. Based on the obtained results, different concentrations of the best carbon and nitrogen sources as well as metal ions for l-asparaginase production were tested. Concentrations of 0.5% w/v glucose, 0.1% w/v ammonium chloride and 10 mM magnesium sulphate were noted to be the optimum concentrations for maximum l-asparaginase production (Fig. 7).

## Discussion

l-Asparaginase has received considerable attention as a primary component in the treatment of acute lymphoblastic leukemia (ALL) (Rati Sinha et al. 2013). Extracellular enzymes have an advantage over the intracellular ones; they could be produced plentifully in the culture medium under normal conditions and could be purified economically (Joseph and Rajan 2011; Vaibhav D Deokar et al. 2010). In this study the extracellular activity was about 305% more than the intracellular one, this offers easy enzyme recovery without the need for cell lysis.

It is reported that bacterial type l-asparaginases are classified into subtypes I and II, which is defined by their intra or extra cellular localization (Michalska and Jaskolski 2006). Type I (cytosolic) has a lower affinity for l-asparagine, while type II (periplasmic) has a high substrate affinity and they also differ in oligomeric form. The periplasmic proteins, known as type II asparaginases (Campbell and Mashburn 1969), from *Escherichia coli* (EcAII) and *Erwinia chrysanthemi* (ErA), have been in clinical use in the treatment of acute lymphoblastic leukemia and some other tumors for more than 30 years (Roberts et al. 1966; Boyse et al. 1967; Bodey et al. 1974; Lay et al. 1975). Accordingly, the present study was focused on extracellular enzyme productivity of the used bacterial isolate. The phylogenetic tree shown in Fig. 1 and Table 3 shows that l-asparaginases of *Bacillus licheniformis* is clustered with those of *Bacillus subtilis* (pairwise distance 0.00267), *Bacillus haloterans* (pairwise distance 0.1128), *Bacillus mojavensis* (pairwise distance 0.11579) and *Bacillus tequilensis* (pairwise distance 0.0892) while it shows distant relatedness to l-asparaginases of other *Bacillus subtilis* species (pairwise distance 2.07678) as well as for those of *Bacillus amyloliquefaciens* and *Bacillus velezensis* species (pairwise distances 2.13201 for each). Figure 2 and Additional file 1: Table S4 show the situation of l-asparaginase for *Bacillus licheniformis* to those of the two FDA approved l-asparaginases of *E. coli* (marketed under the brand name Elspar) and *Erwinia chrysanthemi* (marketed under the brand name Erwinaze), both were used as two reference enzymes. The results reveal that occurrence of *Bacillus licheniformis* l-asparaginase is a cluster distinct from those of the two other reference enzymes (Fig. 2) and a pairwise distances of 1.253 and 1.161 for l-asparaginases of *E. coli* and *Erwinia chrysanthemi*, respectively (Additional file 1: Table S4). Prediction of antigenic determinants (epitopes) along the amino acid sequences of the corresponding l-asparaginases of *E. coli*, *Erwinia chrysanthemi* (as two reference strains for FDA approved l-asparaginases) and *Bacillus licheniformis* showed in between antigenic regions number for *Bacillus licheniformis*. The validity of this prediction is supported by the observation that *Erwinia* asparaginase has less immunogenic associated toxicity as compared to that of *E. coli* asparaginase (Barry et al. 2007). Also Cavanna et al. (1976) reported that l-asparaginase from *E. coli* has more immuno-depressive and immuno-toxic potential than that from *E. carotovora*. The therapeutic effect of l-asparaginases from these two bacterial species (*E. coli* and *Erwinia*) is accompanied by side effects which are partially attributed to the immunogenicity of these enzymes. Comparable number of antigenic regions detected in l-asparaginase of *Bacillus licheniformis* suggests fewer side effects and this could introduce such enzyme source as a potential candidate for therapeutic and medical application.

Regarding enzyme thermal stability, a remarkable stability was demonstrated as the produced enzyme could preserve 80% of its activity after exposure to temperature 70 °C for 30 min. This stability level suits its use medically since higher thermal stability is usually required for industrial enzymes. Our results agreed with a previous report (Elshafei et al. 2012), where l-asparaginase produced from *Penicillium brevicompactum* was stable over wide range of temperatures till 70 °C. The activity of the enzyme preparation of the test isolate increased gradually over the range of 20 up to 35 °C and decreased at 45 °C by 27.7%. It showed maximum activity at 40 °C. The optimum alkaline pH of the enzyme is attributed to that the aspartate liberated by asparagine hydrolysis has lower affinity to the active catalytic site of the enzyme. This enables more binding of asparagine to the enzyme. On the other hand at acidic pH the breakdown of asparagine by the enzyme results in the production of aspartic acid which has high affinity to the enzyme catalytic site, disabling the binding of asparagine to the enzyme (El-Sabbagh et al. 2013). The same results were recorded by other researchers (Elshafei et al. 2012; El-Sabbagh et al. 2013) who found the maximum enzyme activity from *Streptomyces halstedii* and *Penicillium brevicompactum* at pH 8.0, while others reported the maximum activity from *B. licheniformis* and *Streptomyces gulbargensis* at pH 9.0 (Amena et al. 2010; Mahajan et al. 2014). Water molecules play a significant role in protein’s biological function by attaching to the surface and entering into the inner part of protein molecules (Persson and Halle 2008). When water activity is affected by drastic conditions, like extreme temperature, pH or high salinity, normally water may limit the enzyme activity. In our study, l-asparaginase showed increased activity (about 48.0%) at 500 mM sodium chloride concentration. The increase in the activity can be explained as that salinity may stimulate loop flexibility in the structure of the enzyme that can be more involved in enzymatic activity. Halophilic enzymes have high negative charges so can be easily dissociated and become more flexible in the presence of sodium chloride (Han et al. 2014). The current results agree with that of some researches (Elshafei et al. 2012; Dash et al. 2016; Shechtman 2013; Han et al. 2014). A gradual increase in the enzyme activity was reported by increasing asparagine concentration followed by slight decrease in the activity at higher concentration. Similar results were also reported previously (El-Mched et al. 2015). This finding may be attributed to the saturation of the active enzymatic sites by the substrate. Metal ions acts as cofactor for binding at the catalytic site of the enzyme. Zinc and iron slightly increased the activity which is the same as reported by some investigators (Han et al. 2014) but not affected by nickel, copper and cobalt as mentioned by others (Moorthy et al. 2010). Conversely, it disagreed with what was reported previously (El-Sabbagh et al. 2013) where zinc inhibited the activity of l-asparaginase.

Gamma ray, used in strain improvement, it causes mutation through breakage of single and double stranded DNA resulting structural changes or oxidation (Huma et al. 2012). In this study, it improved l-asparaginase production by 1.4 folds compared to the wild type strain. Many researches supported the use of gamma radiation in increasing the enzymes production (Hoe et al. 2016; Huma et al. 2012; Hyster and Ward 2016; Diep et al. 2017).

The RSM includes a group of statistical and mathematical techniques for development of a sufficient relationship between a response of interest and number of variables through conducting some preliminary studies to determine the optimum range for each factor to be used in RSM. Maximum l-asparaginase production occurred at 37 °C. Any decrease or increase from the optimum temperature slows down the metabolic activity of the enzymes as reported by some investigators (El-Hefnawy et al. 2015; Jayaramu et al. 2010). However, Prakasham et al. reported progressive increase in activity by increasing temperature and optimum temperature attained at 39 °C (Prakasham et al. 2007). Our results agreed with what was reported previously (Bahrani 2016). Optimum pH was 7 followed by slight decline in productivity at higher pH values, this may be due to partial enzyme denaturation in response to dissociation of ionizable groups of the enzyme. A significant decrease in enzyme productivity at low pH may be attributed to inhibition of substrate binding to the enzyme as a result of change in the properties and shape of the enzyme and/or the substrate (El-Hefnawy et al. 2015). This agreed with some studies (Kavitha and Vijayalakshmi 2010; Bahrani 2016) but also differed with others (Pradhan and Dash 2013; Jayaramu et al. 2010; Prakasham et al. 2007), where their optimum reported pHs were 6.5, 7.5 and 6, respectively. The importance of incubation time was recorded by some researchers (Pradhan and Dash 2013; Maysa et al. 2010), who mentioned similar results to that of the current study. At prolonged incubation time the production level started to decrease, long incubation time can lead to degradation of the enzyme by the proteolytic enzymes, and also it causes depletion of medium components or production of some enzyme inhibitors in the medium. The shorter incubation time is cost effective and reduces the chance of the enzyme decomposition (El-Hefnawy et al. 2015). On contrary, other studies demonstrated diverse results; the optimum incubation time for *Emericella nidulans* and *Stenotrophomonas maltophilia* was 48 h (El-Mched et al. 2014; Jayaramu et al. 2010), 72 h for *Streptomyces tendae* and *Penicillium oxalicum* (Kavitha and Vijayalakshmi 2010; El-Hefnawy et al. 2015). Owing to the agitation influence on the availability of the oxygen and nutrient in the medium (Sooch and Kauldhar 2013), the increase in the agitation rate helps in mixing of the nutrients which enhance its absorption by the microorganisms (Pansuriya and Singhal 2011), and the decrease in the production at higher agitation rate may be attributed to the shear stress on the bacterial cells (Sooch and Kauldhar 2013). Other published reports revealed higher enzyme production at 220 rpm (Bahrani 2016) and at 150 rpm (Sooch and Kauldhar 2013).

The RSM model has a second order polynomial equation that relates the square root values of l-asparaginase activity to the tested variables. A model of high significance was obtained, as evident from the Fisher’s F-test, with a very low probability value (P model > F) = 0.0001. The goodness of fit of the model was checked by several statistical criteria. The high determination coefficient indicates that only 4.91% of the total variation is not explained by the model. The lack of fit F-value of 19.23 and p-value of 0.0504 imply that lack of fit is not significant relative to the pure error and the model accordingly shows excellent fit. The adjusted R^2^ meant that the model can explain 90.92% of the variability if the sample was a subset of the population other than the studied sample, its value is always lower than the R^2^. The Predicted R^2^ of the model explained that 76.0% of observations other than the fed in data can be correctly anticipated by the model and the model has high predictive abilities (Frost 2013). The model adequately compares the range of the predicted values at the design points to the average prediction error. Ratios greater than 4 indicate adequate model discrimination and it can navigate the design space. Reliability is defined as the overall consistency of measure. Its index measure is the coefficient of variation (CV) which is the ratio of the standard deviation to the mean. The lower the value of the CV, the more reliable and precise the model is considered. It implies high reliability and excellent precision (Shechtman 2013). All of these considerations indicate the adequacy of the established regression model.

The results of the 3D response surface and 2D contour plots revealed that increasing the medium pH to 7.5, shifting the incubation temperature to 40 °C, increasing the agitation rate to 190 rpm and decreasing the incubation time to 18 h increase l-asparaginase productivity by the test isolate.

Regarding media components, glucose has been responsible for bacterial catabolic repression as it is considered as a quickly metabolized substance but in some cases its incorporation help in enhancing the metabolite production. In our study, glucose increased l-asparaginase productivity. Glucose may provide a positive effect on enhancing l-asparaginase biosynthesis (Baskar and Renganathan 2011; El-Hefnawy et al. 2015). Similarly, glucose enhanced l-asparaginase productivity from *Aeromonas* sp., and *Aspergillus terreus* (Amena et al. 2010; Baskar and Renganathan 2011; El-Hefnawy et al. 2015; Doriya and Kumar 2016; Varalakshmi and Raju 2013), while sucrose and sorbitol increased the enzyme biosynthesis from *Streptomyces tendae* (Kavitha and Vijayalakshmi 2010) and lactose in case of *E. coli* (Bahrani 2016). Nitrogen sources help in the production of nucleic acid, protein and cell wall and it affects enzymes production. Ammonium chloride was the best source and it was mentioned in many reports (Baskar and Renganathan 2009; Meghavarnam and Janakiraman 2015; Hymavathi et al. 2010). The optimum concentration for maximum production was 0.1% w/v in our study and it was 0.5% and 2% w/v in other studies (Kavitha and Vijayalakshmi 2010; Amena et al. 2010; El-Hefnawy et al. 2015). Magnesium increased l-asparaginase production, as published previously. In this study, 10 mM magnesium (1.2% w/v) proved to be optimum concentration for l-asparaginase production and a significant decrease in enzyme production occurred at higher concentrations as magnesium may interfere with the bacterial cell division at high concentrations. Based on the above discussion, *Bacillus licheniformis* revealed a promising l-asparaginase production for different applications. Model-based optimization for enzyme production was established in this study and it can be exploited for enzyme production at a large scale level.

## Additional file

**Additional file 1: Table S1.** Levels of reaction conditions of process parameters as independent variables studied in RSM experimental design for optimization of l-asparaginase production by the selected test mutant. **Table S2.** Experiments that were deduced by the RSM experimental design and performed for l-asparaginase production by the mutant. **Table S3.** Pairwise distances among l-asparaginases of bacterial species presented in the phylogenetic tree shown in Fig. 1. **Table S4.** Pairwise distances among l-asparaginases of *Bacillus licheniformis*, *E. coli* and *Erwinia chrysanthemi* presented in the phylogenetic tree shown in Figure 2. **Table S5.** ANOVA of the quadratic model for the process parameters optimization of l-asparaginase productivity by *Bacillus licheniformis* mutant using Box–Behnken central composite design.
