# Supplementary material for: Experimental and bioinformatics study for production of l-asparaginase from Bacillus licheniformis: a promising enzyme for medical application
Source: AMB Express. 2019 Mar 21;9:39. doi: 10.1186/s13568-019-0751-3 (PMC6428875; doi:10.1186/s13568-019-0751-3)
Supplement: Supplementary file 1 — Additional file 1: Table S1. Levels of reaction conditions of process parameters as independent variables studied in RSM experimental design for optimization of l-asparaginase production by the selected test mutant. Table S2. Experiments that were deduced by the RSM experimental design and performed for l-asparaginase production by the mutant. Table S3. Pairwise distances among l-asparaginases of bacterial species presented in the phylogenetic tree shown in Fig. 1. Table S4. Pairwise distances among l-asparaginases of Bacillus licheniformis, E. coli and Erwinia chrysanthemi presented in the phylogenetic tree shown in Figure 2. Table S5. ANOVA of the quadratic model for the process parameters optimization of l-asparaginase productivity by Bacillus licheniformis mutant using Box–Behnken central composite design. [file 13568_2019_751_MOESM1_ESM.docx]

**AMB Express**

**Experimental and bioinformatics study for production of L-asparaginase from *Bacillus licheniformis*: a promising enzyme for medical application**

**Nada A. Abdelrazek^1^, Walid F. Elkhatib^2,3*^,** **Marwa M. Raafat^1^, Mohammad M. Aboulwafa^2*^**

^1^Department of Microbiology and Immunology, Faculty of Pharmaceutical Sciences and Pharmaceutical Industries, Future University, Cairo, Egypt

^2^Department of Microbiology and Immunology, Faculty of Pharmacy, Ain Shams University, African Union Organization St. Abbassia, Cairo 11566, Egypt

^3^Department of Microbiology and Immunology, School of Pharmacy & Pharmaceutical Industries, Badr University in Cairo (BUC), Entertainment Area, Badr City, Cairo, Egypt

***Correspondence: Prof. Dr. Walid F. Elkhatib**

Postal address: Department of Microbiology & Immunology, Faculty of Pharmacy, Ain Shams University, African Union Organization St. Abbassia, Cairo 11566, Egypt

Tel: +202-24051120, Fax: +202-24051107

Email: [walid-elkhatib@pharma.asu.edu.eg](mailto:walid-elkhatib@pharma.asu.edu.eg); [walid2005faisal@yahoo.com](mailto:walid2005faisal@yahoo.com)

ORCID: 0000-0001-5815-3200

**Prof. Dr. Mohammad M. Aboulwafa**

Postal address: Department of Microbiology & Immunology, Faculty of Pharmacy, Ain Shams University, African Union Organization St. Abbassia, Cairo 11566, Egypt

Tel: +202-24051120, Fax: +202-24051107

E-mail: [mohamed_aboualwafa@pharm.asu.edu.eg](mailto:mohamed_aboualwafa@pharm.asu.edu.eg); [maboulwafa@yahoo.com](mailto:maboulwafa@yahoo.com)

ORCID: 0000-0002-0828-1420

**Dr. Marwa M. Raafat**

Department of Microbiology & Immunology, Faculty of Pharmaceutical Sciences and Pharmaceutical Industries, Future University in Egypt.

E-mail: [Marwa.Mahmoud@fue.edu.eg](mailto:Marwa.Mahmoud@fue.edu.eg); [marwrft@yahoo.com](mailto:marwrft@yahoo.com)

ORCID: 0000-0001-5614-5127

**Nada A. Abdelrazek**

Department of Microbiology & Immunology, Faculty of Pharmaceutical Sciences and Pharmaceutical Industries, Future University in Egypt.

E-mail: [Nada.Anwar@fue.edu.eg](mailto:Nada.Anwar@fue.edu.eg); n_s1986@hotmail.com

ORCID: 0000-0001-8082-2298

**Table S1: Levels of reaction conditions of process parameters as independent variables studied in RSM experimental design for optimization of L-asparaginase production by the selected test mutant.**

| **Test variable** | **Variable code** | **Variables levels** |
| --- | --- | --- |
| **Incubation temperature (°C)** | **A** | **-1 0 +1** |
|  |  | **35 37.5 40** |
| **Initial pH** | **B** | **6 7 8** |
| **Incubation time (h)** | **C** | **18 33 48** |
| **Agitation (RPM)** | **D** | **150 175 200** |

**Table S2: Experiments that were deduced by the RSM experimental design and performed for L-asparaginase production by the mutant**

| **Experiment** | **Temperature (°C)** | | **pH** | | **Time (h)** | | **Agitation (rpm)** | |
| --- | --- | --- | --- | --- | --- | --- | --- | --- |
|  | **Value** | **Level code** | **Value** | **Level code** | **Value** | **Level code** | **Value** | **Level code** |
| 1 | 40 | 1 | 7 | 0 | 48 | 1 | 175 | 0 |
| 2 | 37.5 | 0 | 8 | 1 | 33 | 0 | 200 | 1 |
| 3 | 37.5 | 0 | 7 | 0 | 48 | 1 | 200 | 1 |
| 4 | 40 | 1 | 8 | 1 | 33 | 0 | 175 | 0 |
| 5 | 37.5 | 0 | 8 | 1 | 48 | 1 | 175 | 0 |
| 6 | 37.5 | 0 | 8 | 1 | 18 | -1 | 175 | 0 |
| 7 | 35 | -1 | 7 | 0 | 33 | 0 | 150 | -1 |
| 8 | 37.5 | 0 | 7 | 0 | 33 | 0 | 175 | 0 |
| 9 | 37.5 | 0 | 7 | 0 | 18 | -1 | 200 | 1 |
| 10 | 35 | -1 | 6 | -1 | 33 | 0 | 175 | 0 |
| 11 | 35 | -1 | 8 | 1 | 33 | 0 | 175 | 0 |
| 12 | 40 | 1 | 7 | 0 | 33 | 0 | 200 | 1 |
| 13 | 37.5 | 0 | 7 | 0 | 48 | 1 | 150 | -1 |
| 14 | 35 | -1 | 7 | 0 | 48 | 1 | 175 | 0 |
| 15 | 37.5 | 0 | 6 | -1 | 48 | 1 | 175 | 0 |
| 16 | 37.5 | 0 | 7 | 0 | 18 | -1 | 150 | -1 |
| 17 | 37.5 | 0 | 8 | 1 | 33 | 0 | 150 | -1 |
| 18 | 40 | 1 | 7 | 0 | 18 | -1 | 175 | 0 |
| 19 | 40 | 1 | 7 | 0 | 33 | 0 | 150 | -1 |
| 20 | 37.5 | 0 | 6 | -1 | 33 | 0 | 150 | -1 |
| 21 | 37.5 | 0 | 7 | 0 | 33 | 0 | 175 | 0 |
| 22 | 35 | -1 | 7 | 0 | 18 | -1 | 175 | 0 |
| 23 | 40 | 1 | 6 | -1 | 33 | 0 | 175 | 0 |
| 24 | 37.5 | 0 | 6 | -1 | 18 | -1 | 175 | 0 |
| 25 | 35 | -1 | 7 | 0 | 33 | 0 | 200 | 1 |
| 26 | 37.5 | 0 | 7 | 0 | 33 | 0 | 175 | 0 |
| 27 | 37.5 | 0 | 6 | -1 | 33 | 0 | 200 | 1 |

**Table S3: Pairwise distances among L-asparaginases of bacterial species presented in the phylogenetic tree shown in Figure 1.**

|  |  | 1 | 2 | 3 | 4 | 5 | 6 | 7 | 8 | 9 |
| --- | --- | --- | --- | --- | --- | --- | --- | --- | --- | --- |
| 1 | L-asparaginase_*Bacillus_subtilis* |  |  |  |  |  |  |  |  |  |
| 2 | MULTISPECIES:_L-asparaginase_*Bacillus* | 0.00267 |  |  |  |  |  |  |  |  |
| 3 | MULTISPECIES:_type_II_asparaginase_ *Bacillus_amyloliquefaciens*_group | 2.54891 | 2.54891 |  |  |  |  |  |  |  |
| 4 | Type_II_asparaginase_*Bacillus_halotolerans* | 2.05573 | 2.05573 | 2.20263 |  |  |  |  |  |  |
| 5 | Type_II_asparaginase_*Bacillus_licheniformis* | 2.07678 | 2.07678 | 2.13201 | 0.11280 |  |  |  |  |  |
| 6 | Type_II_asparaginase_*Bacillus_mojavensis* | 2.03511 | 2.03511 | 2.25264 | 0.01613 | 0.11579 |  |  |  |  |
| 7 | Type_II_asparaginase_*Bacillus_subtilis*_subsp._spizizenii. | 0.00535 | 0.00803 | 2.54891 | 2.05573 | 2.07678 | 2.03511 |  |  |  |
| 8 | Type_II_asparaginase_*Bacillus_subtilis* | 2.07678 | 2.07678 | 2.13201 | 0.10981 | 0.00267 | 0.11280 | 2.07678 |  |  |
| 9 | Type_II_asparaginase_*Bacillus_tequilensis* | 2.05573 | 2.05573 | 2.15500 | 0.13391 | 0.08920 | 0.14003 | 2.05573 | 0.08628 |  |
| 10 | Type_II_asparaginase_*Bacillus_velezensis* | 2.48221 | 2.48221 | 0.01905 | 2.15500 | 2.13201 | 2.20263 | 2.48221 | 2.13201 | 2.15500 |

**Table S4: Pairwise distances among L-asparaginases of *Bacillus licheniformis*, *E. coli* and *Erwinia chrysanthemi* presented in the phylogenetic tree shown in Figure 2.**

|  |  | 1 | 2 | 3 |
| --- | --- | --- | --- | --- |
| 1 | ansB_*Escherichia_coli*_B354 |  |  |  |
| 2 | Lasparaginase_Dickeya_(formerly_*Erwinia*)_*chrysanthemi* | 0.732 |  |  |
| 3 | L-asparaginase_*Bacillus_licheniformis* | 1.253 | 1.161 |  |

**Table S5: ANOVA of the quadratic model for the process parameters optimization of** **L-asparaginase productivity by *Bacillus licheniformis***
**mutant using Box-Behnken central composite design**

| **Source** | **SS** | | **DF** | **MS** | | **F-value** | **p-value** | | **Comments** | | |
| --- | --- | --- | --- | --- | --- | --- | --- | --- | --- | --- | --- |
| **Model** | 0.34 | | 14 | 0.024 | | 19.59 | <0.0001 | | Significant | | |
| **Residual** | 0.015 | | 12 | 1.223E-003 | |  |  | |  | | |
| **Lack of Fit** | 0.015 | | 10 | 1.453E-003 | | 19.23 | 0.0504 | | not significant | | |
| **Total Regression** | 0.35 | | 26 |  | |  |  | |  | | |
| **Analysis reliability parameters** | | | | | | | | | | | |
| **R-Squared** | | 0.9581 | |  |  | | |  | |  |  |
| **Adjusted R-Squared** | | 0.9092 | |  |  | | |  | |  |  |
| **Predicted R-Squared** | | 0.7600 | |  |  | | |  | |  |  |
| **Adequate Precision** | | 15.913 | |  |  | | |  | |  |  |

^*^Where, SS, Sum of Squares; DF, Degrees of Freedom; MS, Mean of Squares.
